# Assessment of energy cost in three winter sports: Alpine skiing, snowboarding, and simulated cross-country skiing

**DOI:** 10.1080/15502783.2026.2722536

**Published:** 2026-09-03

**Authors:** Lu Li, Zhiqiang Liang, Bo Sun, Chu Yun, Barbara E. Ainsworth, Yu Liu

**Affiliations:** a School of Exercise and Health, Shanghai University of Sport, Shanghai, China; b School of Physical Education, Liaocheng University, Liaocheng, China; c Laboratory of Exercise and Health Sciences of Ministry of Education, Shanghai University of Sport, Shanghai, China

**Keywords:** Olympic sports, winter sports, physical activity, energy costs, METs

## Abstract

**Background:**

Little is known about the energy costs of recreational skiing, snowboarding, and cross-country skiing activities in young adults. Investigating the intensity of such activities for promoting skiing in public health and exercise contexts is needed. This study measured the heart rate (HR), oxygen uptake (VO_2_), and respiratory quotient (RQ) of recreational Alpine skiing, snowboarding, and simulated cross-country skiing (roller skiing) in young adults (17–25 y).

**Methods:**

Three cross-sectional studies were performed. Alpine skiing (study 1, *n* = 13, 11 males, 2 females) and snowboarding (study 2, *n* = 13, all males) were performed on a ski slope rated intermediate in difficulty. Each skier completed one run and was guided by a researcher to maintain a common speed. Simulated cross-country skiing (study 3, *n* = 13, 8 males, 5 females) was performed while the roller skiing on a laboratory treadmill at 0% incline with increasing velocities per stage. Two trials were conducted: free skate (combined V1, V2) and the classic technique. HR was monitored by telemetry. VO_2_ and RQ were measured by portable indirect calorimetry. METs were computed as VO_2_ ml.kg^−1.^min^−1^ divided by 3.5.

**Results:**

The energy cost in METS for men and women combined was highest for simulated cross-country skiing (free skate, 10.57 ± 0.53; classic, 8.62 ± 0.75) than Alpine skiing (7.76 ± 1.18) and snowboarding (7.38 ± 2.55). RQ was highest for Alpine skiing (0.96 ± 0.10) than other skiing modes (on average, 0.87 to 0.88). Exercise HR was higher in the free skate (164.52 ± 13.58) and Alpine skiing (153.41 ± 20.54) than classic skiing (149.56 ± 12.68) and snowboarding (139.39 ± 41.96).

**Conclusions:**

Recreational Alpine skiing, snowboarding, and simulated cross-country skiing are vigorous-intensity activities with an energy cost exceeding 6 METs in young adults. This study provides empirical, protocol-specific MET values for these activities, which validated and refined the existing estimates for these activities in the Adult Compendium of Physical Activities, offering an empirical basis for more precise energy expenditure assessment in recreational skiing.

## 1. Introduction

Prior to 2000, few Chinese youth and adults participated in winter sport activities of Alpine skiing, cross-country skiing, and snowboarding. However, with the anticipation of the 2022 XXIV Olympic Winter Games scheduled to take place in cities and provinces in and around Beijing, China, skiing activities are gaining popularity. In 2019, nearly 13 million Chinese adults and youth visited ski areas in China, a 62% increase since 2014 [1]. Most popular among adults 20 to 50 years of age, skiing is increasing in popularity among Chinese adolescents [1,2].

Skiing and snowboarding are health-enhancing activities consistent with the World Health Organization's (WHO) guidelines to undertake regular physical activity, engage in 150 min per week of moderate-intensity physical activity or 75 min per week of vigorous-intensity activity or a combination of the two intensities, and do muscle-strengthening activities of all major muscle groups at least two days a week [3,4]. Alpine skiing is reported to increase bone mineral density [5,6], reduce systemic hypertension [7] and body fat [8,9], improve leg muscle strength and power [10,11], balance [12], and subjective well-being [13].

According to the 2011 Adult Compendium of Physical Activities [14] (abbreviated as the Adult Compendium) Alpine skiing, snowboarding, cross-country skiing, and roller skiing range in intensity from 4.3 to 15.0 METs, meeting the intensity definitions as moderate- (3.0–5.9 METs) and vigorous-intensity physical activities (≥6.0 METs). A MET is an abbreviation for metabolic equivalent of a task and is defined as an activity metabolic rate divided by a resting metabolic rate.

Studies published on the energy costs of Alpine- and cross-country skiing vary in terms of the skiing venues, skier characteristics, and purpose of skiing tasks. Alpine skiing studies include ski racing [15–20], recreational skiing in older adults [10,21,22], and training studies [4]. Cross-country skiing studies vary by terrain to include skiing on snow [23], roller skiing on laboratory treadmills [24,25], roller skiing outdoors on a flat track [26,27], and roller skiing outdoors in a course designed to simulate a World Cup event [28]. One study evaluated the energy cost of recreational Alpine skiing in younger adults [29], and another has reported the energy cost of snowboarding [30,31]. In comparison of different skiing styles, one study has measured the energy costs of Alpine skiing vs. cross-country skiing [29], and two studies have measured the energy costs of different cross-country skating techniques [23,25]. Cross-country skating techniques included free skate and classic technique. In free skate, there are no restrictions on the athlete's pushing movements, and the skis are not confined to the ski tracks. The classic technique includes double poling, diagonal stride, herringbone (which involves stepping without a glide phase), turning, and downhill skiing, where skating with either one or both skis is prohibited. No studies have compared the energy costs of cross-country free skate and classic (diagonal stride) techniques.

Many factors influence the energy costs of skiing to include differences in study designs and methods used to measure the energy costs of an activity, the environment and characteristics of the skiing surface, the selection of subjects for testing, and the age, fitness, and skiing skills of the research participants [32–34]. Accordingly, it is difficult to generalize the energy costs of skiing and snowboarding activities. The 2011 Adult Compendium provides a resource to identify average MET values of physical activities published in the scientific literature. For example, an Alpine skiing entry in the Adult Compendium, skiing, downhill, Alpine or snowboarding, light effort, and active time only (code 19150), has a MET value of 4.3. This value is averaged from five Alpine skiing measures published in two papers [21,35]. In total, the Adult Compendium contains four entries for Alpine skiing (codes 19075, 19150, 19160, 19170), with an energy cost ranging from 4.3 to 8.0 METs. Owing to a paucity of research studies, MET values for snowboarding are combined with the Alpine skiing entries. Cross-country skiing has six entries (codes 19080–91935) ranging from 6.8 METs for slow skiing to 15.5 METs for ski racing. Roller skiing has one entry (19175) listed at 12.5 METs. Some MET values published in the Adult Compendium have no literature sources, thus are estimated based on the energy costs of similar activities obtained from published studies. For example, the MET value for vigorous-intensity recreational Alpine skiing is estimated at 8 METs, as no studies were found in the literature for this activity.

However, these estimates are often derived from older studies or broad classifications and may not fully capture the physiological responses associated with modern equipment, technical evolutions (such as the combined use of V1 and V2 freestyle techniques), and varying intensities. Therefore, contemporary empirical validation of these estimates is warranted. Furthermore, refining the existing data through protocol-specific measurements (e.g. across different grades, velocities, and techniques) is necessary to enhance their precision and applicability in sports science, rehabilitation, and public health. The purpose of this study was to measure the energy cost of three Olympic winter sport activities: Alpine skiing, snowboarding, and cross-country skiing (simulated as roller skiing), in research settings. Alpine skiing and snowboarding were performed on a downhill ski course, and cross-country skiing was simulated using roller skiing techniques on a laboratory treadmill.

## 2. Materials and measures

### 2.1. Study design and setting

Three independent cross-sectional studies were performed to assess the energy cost of Alpine skiing (study 1), snowboarding (study 2), and treadmill roller skiing, herein referred to as simulated cross-country skiing (study 3). Data were collected from March to April 2021 at the Sun Ski Resort located in Heilongjiang Province, China (Alpine skiing and snowboarding), and at the Shanghai University of Sport in Shanghai, China (simulated cross-country skiing). The materials used for data collection were the same for each study. The study protocols were approved by the Institutional Review Boards at Harbin Sports University and the Shanghai University of Sport. All the subjects read and signed an informed consent form prior to data collection.

### 2.2. Materials

The participants' age in years and skiing or snowboarding experience in years were measured using a questionnaire developed for this study. Height in centimeters (cm) and weight in kilograms (kg) were measured using standard laboratory scales. The heart rate in beats per minute (HR, b.min^−1^) during rest and exercise was measured using a Garmin heart-rate monitor (Garmin HRM-Dual, Schaffhausen, Switzerland) equipped with an elastic strap worn around the chest to record each heartbeat. Oxygen uptake in absolute (milliliters per minute, VO_2_ ml.min^−1^) and relative terms (milliliters per kilogram body weight, VO_2_ ml.kg.min^−1^) and the respiratory quotient (RQ) were obtained with the COSMED K5 wearable metabolic system (COSMED, Rome, Italy). The K5 unit weighs 900 g and is attached to a shoulder harness that allows the unit to rest on the participant's upper back. Inspired air is collected by the K5 face mask worn over the participant's mouth and nose. The volume of inspired air and fractions of O_2_ and CO_2_ in the expired air were analyzed by the K5 unit for calculation of VO_2_ in ml.min^−1^ and RQ. The K5 system was set to mixing chamber mode, with VO_2_ data calculated every 10 s during rest and exercise to provide more stable airflow data compared to breath-by-breath mode for all three skiing tests [36]. The metabolic data were recorded by the K5 unit during the Alpine skiing and snowboarding session, with the start and end points of exercise marked by the investigator. Each participant wore their own equipment to complete their respective Alpine skiing, snowboarding, and simulated cross-country skiing sessions. Simulated cross-country skiing was performed using roller skis on a laboratory treadmill (Roddy RL3500E Skating Treadmill, Hagby, Sweden).

### 2.3. Collection and preparation of study data

The data are presented as the rest and exercise HR, VO_2_, RQ, and METs for each study. HR and VO_2_were collected during 2 min of rest before exercise and continuously during each exercise session. HR and VO_2_ data were downloaded from the Garmin and K5 systems, respectively, and stored in a laptop computer after each participant completed their exercise session. VO_2_ was collected in milliliters (ml) and converted to L.kg.min^−1^ with the formula: (VO_2_ ml × 1000)/weight kg/time min. Heart rate was collected as beat-by-beat and summed as b.min^−1^ METs were computed as VO_2_ ml.kg.min^−1^ divided by 3.5 ml.kg.min^−1^. A standard value of 3.5 ml.kg.min^−1^ was used as the resting metabolic rate to be consistent with the MET values presented in the Adult Compendium [14].

## 3. Methods

### 3.1. Alpine skiing and snowboarding

A total of 26 junior skiers from Harbin Sports University volunteered to participate in studies 1 and 2. The participants ranged in age from 19 to 23 years and had 0.5–4 years of skiing and snowboarding experience. Among the participants, 13 were specialists in Alpine skiing (11 males and 2 females), and 13 were specialist in snowboarding (13 males, 0 females). Descriptive information for all the participants is presented in Table 1.

**Table 1.** Characteristics of study participants in Alpine skiing, snowboarding, and simulated cross-country skiing (treadmill roller skiing).

Sport	Sex	N	Age (years) (m ± sd)	Skiing experience (years) (m ± sd)	Height (cm) (m ± sd)	Weight (kg) (m ± sd)
Alpine skiing	Male	11	20.64 ± 1.17	1.41 ± 0.78	182.09 ± 4.30	78.09 ± 8.77
	Female	2	20.50 ± 0.50	2.75 ± 1.25	175.50 ± 5.50	61.75 ± 1.25
	All	13	20.69 ± 1.19	1.65 ± 0.76	181.08 ± 5.51	75.58 ± 9.91
Snowboarding^a^	Male	13	20.85 ± 0.68	1.23 ± 0.44	176.77 ± 3.85	72.31 ± 8.21
Simulated cross-country skiing	Male	7	21.00 ± 2.67	6.75 ± 2.43	176.88 ± 5.72	71.06 ± 6.65
	Female	5	19.00 ± 2.00	7.40 ± 1.14	168.80 ± 2.95	57.40 ± 5.77
	All	12	20.23 ± 2.55	7.00 ± 2.00	173.77 ± 6.22	65.81 ± 9.21

^a^ No females were recruited in snowboarding.

m = mean.

sd = standard deviation.

**Figure 1.**
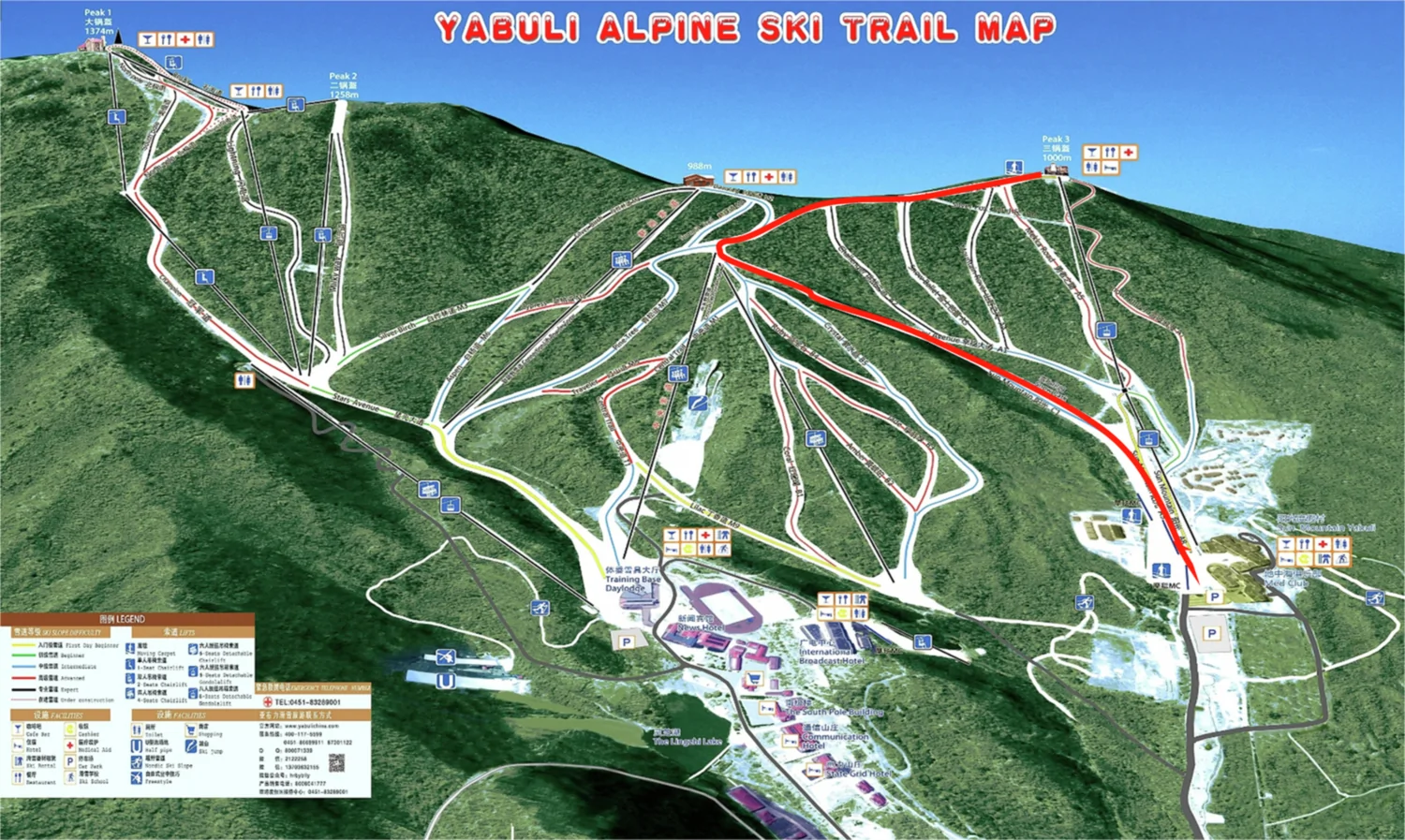
Skiing path of metabolic test for Alpine skiing and snowboarding skiing (red line is skiing path).

The Alpine skiing and snowboarding studies were performed at Sun Mountain Yabuli Ski Resort located in Harbin, Heilongjiang Province. Providing 17 slopes, the ski area is located at 1499–4508 feet above sea level. The slope selected for the trial is classified as intermediate and has a distance of 3 km to 3.7 km and a maximum drop of 540 m from start to end. The ski run is marked with the red line in Figure 1.

Alpine skiing and snowboarding followed the same protocol. Data were collected from March 27th to 30th, 2021. The ambient temperature during the skiing trials ranged from 6 °C to −5 °C. Prior to data collection, the participants were asked to refrain from eating food and performing high-intensity exercise for two hours before their trial. Trials started in the ski lodge lobby where participants read and signed an informed consent form, assembled their skiing and snowboarding equipment, filled out a questionnaire for their age and years of skiing experience, were informed of the experimental protocol, including the requirements of the skiing and snowboarding techniques, and were given a picture of the ski course to view. Height and weight were measured prior to the participants being fitted with the heart rate monitor and K5 equipment. To ensure that all the participants skied on the correct slope and at a moderate recreational speed, which was recorded by the GPS, two participants skied together and were navigated by an elite skier down the slope to make sure they could finish within 40 min, which represented typical recreational downhill skiing intensity. The speed in the Alpine skiing group was 10.05 ± 2.50 km/h, and that in the snowboarding group was 12.00 ± 2.42 km/h. All the participants performed warm-up exercises for 3 to 5 min after arriving at the top of the ski run. Resting HR and VO_2_ were recorded for two minutes prior to each exercise session. Exercise data were collected continuously, with the average VO_2_ recorded every 30 s. Skiers completed the ski run from start to end in about 7 to 10 min.

### 3.2. Simulated cross-country skiing

A total of 12 senior-level cross-country skiers (aged 17–25 years) with 4–9 years of competitive cross-country skiing were recruited from skiers training on teams in Jilin Province in Northeast China. Upon recruitment, participants identified the number of years they had engaged in cross-country skiing and their ability to use roller skis on a treadmill. Anthropometric and descriptive information for the cross-country skiers is described in Table 1.

The study was conducted in the Biomechanical Laboratory at the Shanghai University of Sport. The participants were asked to refrain from eating food and exercising vigorously for at least two hours before the testing sessions. The testing sessions started with the participants reading and signing an informed consent form and having their height (cm) and weight (kg) measured using laboratory scales. They were informed of the testing protocol and allowed to familiarize themselves with roller skiing on a treadmill before the testing protocol. After the warm up, the researchers attached the HR monitor and the K5 system to the participants with instructions to sit in a resting condition for 5 min to obtain resting HR and VO_2_ values.

**Figure 2.**
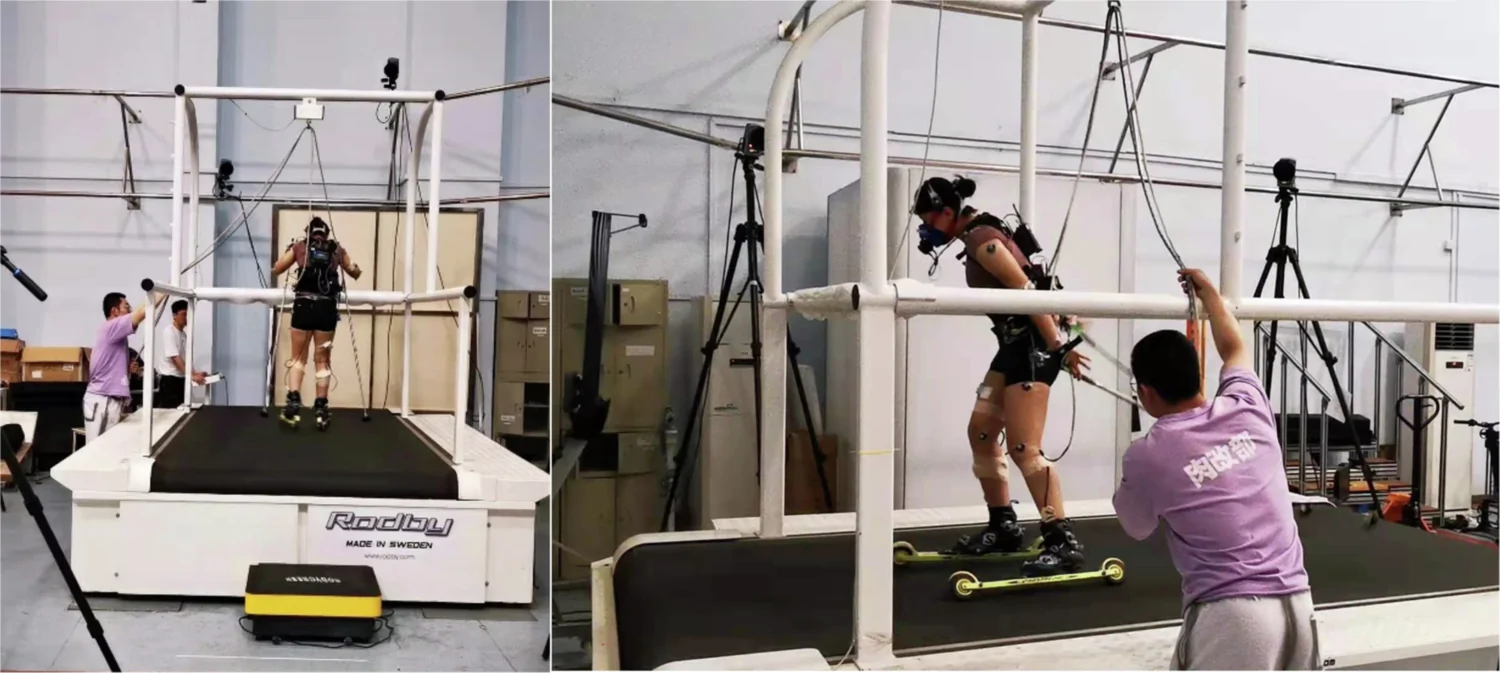
The experimental environment for metabolic test of cross-country skiing.

The participants completed two simulated cross-country ski tests with randomized orders: one using freestyle V1 and V2 skate techniques interchangeably and one using the classic (diagonal stride) technique. The freestyle V1 skate is primarily an uphill technique and involves having three points of contact (two poles and one ski) in each stroke. The freestyle V2 skate is primarily for flat terrain and involves a double pole push with every skate. The classic ski technique is for uphill and flat terrain and consists of pushing the arm and leg diagonally with a kicking and gliding motion. The skate graded exercise treadmill test consisted of 5 × 3-min stages at 0% incline, with the velocity increasing by 1.5 km.h^−1^ at each stage. The freestyle technique test was completed as follows: during each stage, the participants were asked to use V1 and V2 skate equally to simulate a mixed-freestyle technique to reflect athletes' technique strategies in actual competitions. The classic-technique test was completed as follows: the classic treadmill graded exercise test consisted of 3 × 3-min stages at 0% incline, with the velocity increasing by 3 km h-1 at each stage. Considering the differences in energy metabolic stability caused by varying peak oxygen uptake demands, different speed increments are set [36]. Figure 2 shows an image of the simulated cross-skiing testing scenario. Table 2 presents the speeds in km.h^−1^ for each graded treadmill ski test for men and women.

**Table 2.** Velocities for each 3-min stage of the graded treadmill simulated cross-country skiing tests for males and females.

Technique	Stage^a^	Velocity km.h^−1^	
Freestyle (V1+V2)		Males	Females
	1	15.0	13.5
	2	16.5	15.0
	3	18.0	16.5
	4	19.5	18.0
	5	21.0	19.5
Classic			
	1	15.0	13.5
	2	18.0	16.5
	3	21.0	19.5

^a^ Each stage was skied at 0% grade.

The treadmill tests were terminated when the skier completed all stages of the test, reached a maximum oxygen consumption (VO_2_ max), could no longer complete the stage, or were stopped by the study investigators for any reason. The criteria for VO_2_ max included a plateau in VO_2_ with an increase in work rate, a RQ > 1.0, or reaching an age-adjusted estimate of HR max computed as 220 minus age [37]. Data were recorded continuously during the tests, with the last two minutes of each stage used to determine the HR and VO_2_ at each stage. All skiers were allowed verbal encouragement from staff and team members during skiing.

### 3.3. Data analysis

Means ± standard deviations were computed for HR b.min^−1^, VO_2_ ml.min^−1^, VO_2_ ml.kg.min^−1^, METs, and RQ with subjects at rest and during Alpine skiing, snowboarding, and simulated cross-country skiing. Owing to the limited sample sizes, analyses were performed for all the subjects combined and male to male between Alpine skiing and snowboarding group to avoid that the result was distorted by gender composition. Independent samples *t*-tests were applied to compare each metabolic parameter among Alpine skiing vs snowboarding. Paired samples *t*-tests were applied to compare each metabolic parameter among freestyle vs classic technique in simulated cross-country skiing. No statistical inferences were made regarding gender differences. Data were analyzed using MATLAB 2018b (The MathWorks, Natick, Massachusetts, USA).

## 4. Results

### 4.1. Descriptive characteristics

Table 1 shows the descriptive data for the study subjects. More males than females participated in Alpine- and simulated cross-country skiing. No females participated in snowboarding. With the exception of Alpine skiers, the subjects were similar in age, skiing experience, height, and body mass. Body height and mass were highest in Alpine skiers.

### 4.2. Alpine skiing and snowboarding

Table 3 shows the average HR, VO_2_ in absolute and relative terms, and MET values for male, female and all skiers combined in the Alpine skiing, snowboarding, and simulated cross-country skiing groups. No significant statistical differences were found in VO_2_, HR, MET and pRQ between all participants and males of Alpine skiing and snowboarding. Resting VO_2_ was similar among all the participants and resting. The peak RQ was 0.96 ± 0.12 for Alpine skiing, and 0.87 ± 0.10 for snowboarding, indicating that skiing activities required a high level of aerobic energy expenditure. On average, the MET values ranged from 6.58 to 8.94 and from 4.83 to 9.93 for Alpine skiing and snowboarding, respectively.

**Table 3.** Means and standard deviations (m ± sd) for oxygen uptake (VO_2_), heart rate (HR), peak respiratory quotient(pRQ), and MET (metabolic equivalent) values during rest and on-snow recreational Alpine skiing and snowboarding and treadmill roller skiing (simulated cross-country skiing). Panel a (males); panel b (females); panel c (all).

			Simulated treadmill cross-country *N* = 7 (m ± sd)			
	Alpine skiing *N* = 11(m ± sd)	Snowboarding *N* = 13(m ± sd)	Free skate		Classic	
a. Males						
Resting						
HR b.min^−1^	84.87 ± 15.24	87.65 ± 10.58	79.78 ± 9.69		79.78 ± 9.69	
VO_2_ ml.min^−1^	449.74 ± 176.32	418.60 ± 74.08	492.36 ± 60.37		492.36 ± 60.37	
VO_2_ ml.kg.min^−1^	5.28 ± 0.88	5.77 ± 0.69	6.99 ± 0.57		6.99 ± 0.57	
METs	1.50 ± 0.25	1.65 ± 0.20	1.99 ± 0.17		1.99 ± 0.17	
Exercise						
HR b.min^−1^	152.1 ± 22.14	139.39 ± 41.96	155.42 ± 12.42		142.7 ± 9.55	
VO_2_ ml.min^−1^	2043.24 ± 436.37	1909.14 ± 645.18	2574.34 ± 340.20		2082.94 ± 364.44	
VO_2_ ml.kg.min^−1^	27.64 ± 4.35	25.87 ± 8.93	36.52 ± 3.10		29.84 ± 3.45	
METs	7.90 ± 1.25	7.38 ± 2.55	10.43 ± 0.88		8.52 ± 0.97	
pRQ	0.97 ± 0.12	0.87 ± 0.10	0.88 ± 0.03		0.86 ± 0.03	
b. Females^a^						
		Simulated treadmill cross-country *N* = 5 (m ± sd)				
	Alpine skiing *N* = 2(m ± sd)	Free skate 1		Classic		
**Resting**						
HR b.min^−1^	114.03 ± 26.27	94.01 ± 15		94.01 ± 15		
VO_2_ ml.min^−1^	656.08 ± 439.6	318.75 ± 123.51		318.75 ± 123.51		
VO_2_ ml.kg.min^−1^	5.52 ± 0.25	5.48 ± 2.04		5.48 ± 2.04		
METs	3.01 ± 1.94	1.57 ± 0.58		1.57 ± 0.58		
**Exercise**						
HR b.min^−1^	160.0 ± 11.31	174.41 ± 7.96		158.8 ± 10.1		
VO_2_ ml.min^−1^	1531.49 ± 166.73	2093.01 ± 162.89		1697.38 ± 155.36		
VO_2_ ml.kg.min^−1^	24.77 ± 1.99	36.59 ± 2.54		29.67 ± 2.51		
METs	7.10 ± 0.56	10.45 ± 0.72		8.47 ± 0.70		
pRQ	0.95 ± 0.04	0.89 ± 0.03		0.89 ± 0.07		
c. All						
				Simulated treadmill cross-country *N* = 12 (m ± sd)		
	Alpine skiing *N* = 13(m ± sd)	Snowboarding^b^ *N* = 13 (m ± sd)		Free skate 1		Classic
**Resting**						
HR b.min^−1^	93.34 ± 18.6	87.65 ± 10.58		85.71 ± 13.66		85.71 ± 13.66
VO_2_ ml.min^−1^	449.74 ± 176.32	418.60 ± 74.08		420.02 ± 124.61		420.02 ± 124.61
VO_2_ ml.kg.min^−1^	5.32 ± 0.8	5.77 ± 0.69		6.36 ± 1.52		6.36 ± 1.52
METs	1.75 ± 0.85	1.65 ± 0.20		1.82 ± 0.43		1.82 ± 0.43
**Exercise**						
HR b.min^−1^	153.41 ± 20.54	139.39 ± 41.96		164.52 ± 13.58		149.56 ± 12.68
VO_2_ ml.min^−1^	2043.24 ± 436.37	1909.14 ± 645.18		2373.78 ± 366.82**		1949.1 ± 363.2
VO_2_ ml.kg.min^−1^	27.16 ± 4.13	25.87 ± 8.93		37.02 ± 1.85**		30.21 ± 2.68
METs	7.76 ± 1.18	7.38 ± 2.55		10.57 ± 0.53**		8.62 ± 0.75
pRQ	0.96 ± 0.10	0.87 ± 0.10		0.88 ± 0.03		0.88 ± 0.05

^a^ Women did not participate in the snowboarding activity.

^b^ Data for male subjects only.

^**^ Indicates significant intergroup differences (*p *< 0.01).

### 4.3. Simulated cross-country skiing

Table 3 shows the HR, VO_2,_ in relative and absolute terms, and MET values for males, females, and all subjects combined in the freestyle skate and classic ski groups. Exercise absolute VO_2_ (t = 5.205, *p* = 0.000) and relative VO_2_ (t = 8.259, *p* = 0.000) and METs (t = 7.031, *p* = 0.000) were significantly higher in the freestyle skate trial than in the classic trial. No significant statistical differences were found in HR and pRQ. The resting VO_2_ values were similar among all the subjects and resting. The MET values were on the order of 7.87–9.37 and 10.04–11.1 for males and females combined in the freestyle skate and classic techniques, respectively.

**Figure 3.**
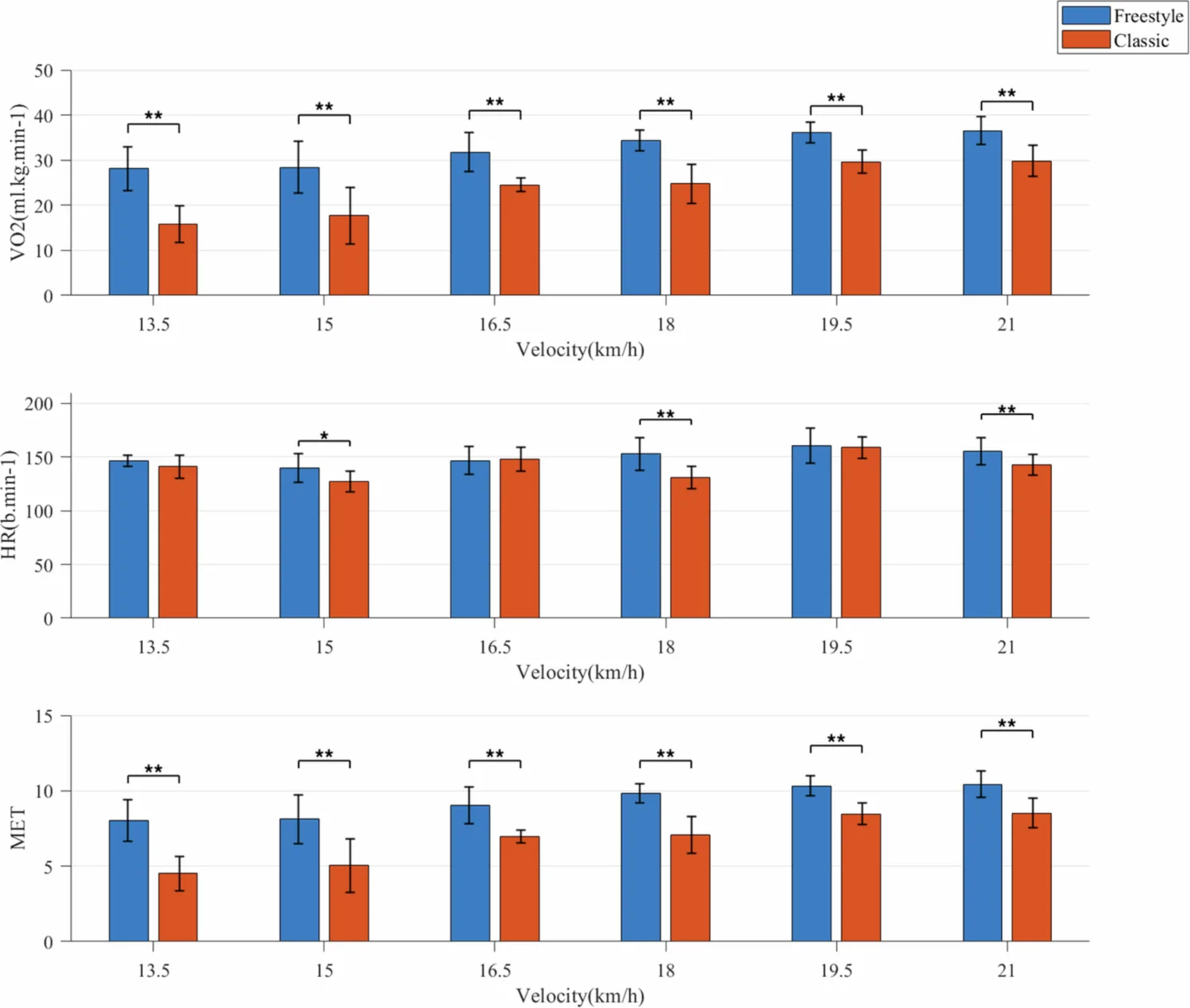
VO_2_, HR, and MET with increasing velocities in freestyle and classic technique. ** indicates significant intergroup differences (*p* < 0.01), * indicates significant intergroup differences (*p* < 0.05).

Table 4 shows the velocity in km.h^−1^, HR in b.min^−1^, VO_2_ in absolute and relative terms, and MET values for males, females, and all subjects combined in the freestyle skate and classic skiing techniques during the graded simulated cross-country skiing tests. There was a graded increase in VO_2_, HR, and MET values with increasing velocities in males and females. Exercise absolute VO_2_, relative VO_2_, HR and METs were significantly higher in freestyle than in classic at 15 km/h, 18 km/h, and 21 km/h. Exercise absolute VO_2_, relative VO_2_ and METs were significantly higher in freestyle than in classic at 13.5 km/h, 16.5 km/h, and 19.5 km/h, while no significant differences were found in HR (Figure 3).

**Table 4.** Means and standard deviations (m ± sd) for energy cost and heart rate during simulated cross-country skiing on a treadmill graded exercise test at 0% grade and increasing velocities at each stage^a^ (*n* = 7 male, *n* = 5 females).

Speed	*N*	Free skate (V1 + V2)				Classic technique			
		Exercise VO_2_ ml	Exercise VO_2_ L.kg.min^−1^	Exercise HR b.min^−1^	Exercise METs	Exercise VO_2_ ml	Exercise VO_2_ ml.kg.min^−1-1^	Exercise HR b.min^−1^	Exercise METs
*13.5 km/h*									
Male	–	–	–	–	–	–	–	–	–
Female	5	1607.49 ± 278.96	28.12 ± 4.90	146.47 ± 5.31	8.03 ± 1.39	919.48 ± 293.91	15.83 ± 4.01	141 ± 10.93	4.53 ± 1.14
All	5	1607.49 ± 278.96	28.12 ± 4.90^**^	146.47 ± 5.31	8.03 ± 1.39^**^	919.48 ± 293.91	15.83 ± 4.01	141 ± 10.93	4.53 ± 1.14
*15 km/h*									
Male	7	1952.93 ± 384.42	27.85 ± 5.37	132.58 ± 11.79	7.96 ± 1.53	1253.61 ± 488.98	17.67 ± 6.22	127.05 ± 9.7	5.04 ± 1.78
Female	5	1650.22 ± 302.38	29.14 ± 6.39	150.8 ± 6.12	8.33 ± 1.81	–	–	–	–
All	12	1826.80 ± 377.56	28.39 ± 5.72^**^	140.17 ± 13.32^*^	8.11 ± 1.63^**^	1253.61 ± 488.98	17.67 ± 6.22	127.05 ± 9.7	5.04 ± 1.78
*16.5 km/h*									
Male	7	2237.03 ± 235.69	32.11 ± 5.59	138.46 ± 9.28	9.12 ± 1.59	–	–	–	–
Female	5	1808.05 ± 149.25	31.21 ± 1.56	158.5 ± 7.18	8.91 ± 0.44	1401.00 ± 131.06	24.54 ± 1.44	148.2 ± 11.34	6.98 ± 0.44
All	12	2058.29 ± 294.60	31.73 ± 4.34^**^	146.81 ± 13.06^**^	9.03 ± 1.23^**^	1401.00 ± 131.06	24.54 ± 1.44	148.2 ± 11.34	6.98 ± 0.44
*18 km/h*									
Male	7	2418.12 ± 274.04	34.46 ± 2.31	143.10 ± 11.86	9.84 ± 0.66	1729.33 ± 261.14	24.74 ± 4.28	130.56 ± 10.63	7.07 ± 1.21
Female	5	1934.55 ± 163.22	34.18 ± 2.26	166.66 ± 6.88	9.76 ± 0.65	–	–	–	–
All	12	2183.86 ± 339.67	34.34 ± 2.24^**^	152.92 ± 15.45^**^	9.81 ± 0.64^**^	1729.33 ± 261.14	24.74 ± 4.28	130.56 ± 10.63	7.07 ± 1.21
*19.5 km/h*									
Male	7	2522.72 ± 186.83	35.83 ± 2.20	149.08 ± 11.13	10.23 ± 0.62	–	–	–	–
Female	5	2093.00 ± 162.89	36.59 ± 2.54	174.41 ± 7.96	10.45 ± 0.72	1697.38 ± 155.36	29.67 ± 2.51	158.8 ± 10.1	8.47 ± 0.70
All	12	2299.86 ± 283.08	36.17 ± 2.33^**^	160.59 ± 16.08	10.33 ± 0.66^**^	1697.38 ± 155.36	29.67 ± 2.51	158.8 ± 10.1	8.47 ± 0.70
*21 km/h*									
Male	7	2574.34 ± 340.20	36.52 ± 3.10	155.42 ± 12.42	10.43 ± 0.88	2128.92 ± 367.73	29.84 ± 3.45	142.7 ± 9.55	8.52 ± 0.97
Female	–	–	–	–	–		–	–	–
All	7	2574.34 ± 340.20	36.52 ± 3.10^**^	155.42 ± 12.42**	10.43 ± 0.88^**^	2128.92 ± 367.73	29.84 ± 3.45	142.7 ± 9.55	8.52 ± 0.97

^a^ Cells with missing values were not part of the graded treadmill test protocol for males and/or females (see Table 2 for the treadmill protocols).

^*^ Indicates significant differences (*p *< 0.05).

^**^ Indicates significant differences (*p *< 0.01).

## 5. Discussion

This study measured the energy costs of recreational Alpine skiing and snowboarding while skiing downhill on a ski slope and during simulated cross-country skiing while roller-skiing on a laboratory treadmill. On average, the MET values during recreational Alpine skiing and snowboarding were slightly higher during Alpine skiing (7.76 ± 1.18) than snowboarding (7.38 ± 2.55). MET values during simulated cross-country skiing during a graded-treadmill test were higher using the mixed V1/V2 freestyle racing-simulation for both males and females (males, 10.43 ± 0.88; females, 10.45 ± 0.72) than the classical diagonal stride trial (males, 8.52 ± 0.97; females, 8.47 ± 0.70). We also found that the energy cost of simulated cross-country skiing increased with increasing treadmill speeds among male and female skiers. According to the MET value classifications for exercise intensities identified by the World Health Organization [3], Alpine skiing, snowboarding, and simulated cross-country skiing are considered vigorous-intensity physical activities (i.e. ≥6 METs).

A significant finding of these studies was the relatively similar VO_2_ for recreational Alpine skiing and snowboarding as compared to simulated cross-country skiing, which was considerably higher. In comparing Alpine skiing with snowboarding in males, the exercise HR was nearly 10 b.min^−1^ faster during Alpine skiing than during snowboarding; also, the RQ was higher in Alpine skiing than in snowboarding; however, these differences were not tested for statistical significance. While both activities were performed on the same intermediate-rated ski hill with subjects being guided down the slope using wide turns to control their velocity to a recreational speed, the skills and techniques required of Alpine skiing and snowboarding differ significantly. For example, Alpine skiing requires the use of poles to keep the upper body stable, which involves a higher percentage of the whole-body musculature (upper-body and lower-body muscle mass), whereas in snowboarding, poles are not used, possibly engaging less muscle mass and energy expenditure to perform the skill. Biomechanically, Alpine skiing and snowboarding require different movements that may also affect the metabolic demands of Alpine skiers and snowboarders [30]. From a biomechanical perspective, this phenomenon could also be explained by the unique motion types causing differences in the implementation of the neuromusculoskeletal system. For example, Alpine skiers should always face the direction of motion during skiing, which engages more muscle mass. When Alpine skiers make a turn, they need a huge eccentric contraction of their low extremity muscle mass to provide enough centripetal force to complete the turn [38]. In contrast, for most of the downhill run, the snowboarder's body position faces toward the vertical direction (upright and sideways) of the motion direction and this limits their muscle participation [39]. Further, Alpine skiers experience more knee and ankle joint torque than snowboarders while completing a turn, which may increase the energy cost of Alpine skiing [40].

To date, few studies have measured the energy cost of Alpine skiing in recreational skiers [21,29] and only one has reported the energy expended in snowboarding [31]. In 2016, Stoggl [29] reported the energy cost of non-racing, Alpine skiing in 11 males and females, mean age 38 ± 4 years as 29.8 ± 0.9 ml. kg. min^−1^ (equal to 8.5 METS). This value is higher than the energy cost of Alpine skiing observed in this study (7.90 METs). The 2011 Adult Compendium estimates vigorous-intensity Alpine skiing as 8.0 METs. Accordingly, the empirically measured Alpine skiing MET value (7.90 METs) in this study closely aligns with this estimate, providing empirical support for the validity and independent empirical evidence for future updates to the Compendium. However, the empirically measured snowboarding MET value (7.38 METs) was lower than that in the Compendium. Considering that there have been few studies designed to measure the MET value of snowboarding, this discrepancy warrants further verification through additional comparative research.

Petrofsky et al. [31] reported the energy expenditure for snowboarding in 8 males, with a mean age of 32.25 ± 7.52 as 1422 ± 170 ml.min^−1^. This value is lower than the value observed in men in this study (1909 ± 645 ml.min^−1^). Unfortunately, Petrofsky's absolute VO_2_ values are not presented as relative VO_2_ values in ml.kg.min^−1^, so it is impossible to determine the MET energy cost of snowboarding from their study. Accordingly, the measured MET value of 7.38 in this study provides the initial MET value for snowboarding.

Another key contribution of this study is the provision of empirical, protocol-specific MET values. The MET ranges measured in the current study (Alpine skiing: 7.90 METs; snowboarding: 7.38 METs; freestyle skiing: 10.43 METs; classic skiing: 8.52 METs) are generally consistent with the estimates in the Adult Compendium of Physical Activities (e.g. 8.0 METs for vigorous-intensity Alpine skiing), providing empirical support for the validity of the latter. Furthermore, this study refines the existing compendium data by revealing specific MET differences across skiing techniques (mixed V1/V2 freestyle vs. classic) and varying speed gradients. These findings offer contemporary empirical evidence for future updates to the Adult Compendium of Physical Activities and may enhance its precision for applications in sports science and public health.

Because of a paucity of snow available to measure the energy costs of cross-country skiing on snow, we measured the energy costs of two simulated cross-country skiing techniques—free skate and classic (diagonal stride style) – on a laboratory treadmill using roller skis. As expected, the energy cost of simulated cross-country skiing was higher than that observed for Alpine skiing and snowboarding, which is consistent with studies published on the energy expenditures of Alpine- and cross-country skiing [29]. The average energy cost of the mixed V1/V2 freestyle skate trial was nearly 2 METS higher than the energy cost for the classic trial for both males (skate, 10.43 ± 0.88; classic, 8.52 ± 0.97) and females (skate, 10.45 ± 0.72; classic, 8.47 ± 0.70). This finding is novel, as no studies have compared the aerobic energy requirements of mixed V1/V2 freestyle skating with classic cross-country skiing while using roller skis on a treadmill graded test. The value of knowing these metabolic results during simulated cross-country skiing performed with different techniques and speeds opens an opportunity to provide precise guidance to competitive skiers with varied skill levels during coaching sessions. For example, knowing the energy cost can aid with tracking the cross-country skiing speed with GPS and/or acceleration sensors such as wearable devices and sport watches during the activity. This information also provides a reliable reference for making an accurate training plan or exercise prescription based on a skier's fitness level.

It is difficult to compare the energy costs of the simulated cross-country skiing trials obtained in this study with studies using different protocols (i.e. snow vs. roller skis), treadmill skiing with different velocities and percent grade patterns (i.e. consistent grade and increasing velocity per stage vs. increasing grades and consistent velocity per stage), and sites (i.e. laboratory treadmill vs. outdoor track). As our protocol used a 0% grade with increasing velocities during the graded treadmill test, the study closest to that condition is that of Millet et al. [27], who recorded a VO_2_ of 44 ml.kg^−1.^min^−1^ (value estimated from a figure) in 13 highly-skilled males during V2 freestyle skating at a velocity of 19.0 km.h^−1^ on an outdoor track. This value is higher than that observed in 8 males in this study completing the freestyle skate stage at 19.5 km.h^−1^ and 0% grade (35.83 ± 2.20 ml.kg^−1.^min^−1^). Several factors may explain the differences between the energy expended in the current study and that in Millet et al. [27] study. First, compared to the study by Millet [27], the participants of present study were less professional. Millet recruited national-level athletes on a real track to measure their energy cost, and we only collected normal skier in a treadmill condition. Second, this research utilized more technologically advanced skiing equipment, special for simulation, which may have assisted athletes in expending less physiological energy, thereby resulting in lower oxygen consumption (VO₂). Third, this study allowed the mixed use of V1 and V2 techniques, whereas Millet's [27] research likely tested only a single technique (V2). This suggests that participants in the current study could select technique combinations with lower metabolic costs based on real-time fatigue levels and speed, thereby optimizing overall efficiency and reducing oxygen consumption per unit of time. Additionally, advances in sports physiology and biomechanics over the past two decades have led to more efficient movement pattern training and energy conservation strategies.

There are several practical applications for the data obtained in this study. First, if a coach, exercise professional, teacher, or parent wanted to know the types of physical activities that are similar in intensity to the skiing activities measuring in this study, he or she one can find this information in the 2011 Adult Compendium. For example, in this study, the MET value for Alpine skiing ranges from 7.76 for males and females combined, and for snowboarding, the MET value was 7.38 for males. Activities similar in intensities include bicycling at 12–13.9 miles per hour (8.0 METs), competitive basketball (8.0 METs), competitive badminton (7.0 METs), tennis (7.3–8.0 METs), and hiking with a day pack with weighted loads of 10–42 pounds (7.3–8.3 METs). The energy costs of simulated cross-country skiing with the freestyle skate and classic techniques for males and females combined in this study measured 10.57 METs and 8.62 METs, respectively. Activities with similar intensities in the Adult Compendium include competitive soccer (10 METs), bicycling at 14 to 15.9 miles.h^−1^ (10 METs), running at 7 miles.h^−1^ (11 METs), and freestyle swimming at 75 yards.m^−1^ (10 METs), running at 5 miles.h^−1^ (8.3 METs), taking a spin cycling class (8.5 METs), stairclimbing (8.0 METs) and hiking and climbing hills (8.3 METs). Second, the data obtained in this study can be a springboard to performing additional research studies of the energy costs of skiing and snowboarding activities in youth, adults, and older persons with different skill levels and fitness categories. Third, as skiing activities gain in popularity, governments and community organizations can promote skiing as a health enhancing activity to be included schools and communities.

The metabolic data reported for recreational Alpine skiing and snowboarding provide additional empirical data on the energy costs of two winter sports activities that are performed globally [1]. When these MET values are added to the Adult Compendium, this information can be accessed by recreational skiers, exercise professionals, and others interested in knowing the energy costs of Alpine skiing and snowboarding. Studies have measured the energy cost of cross-country skiing on snow [20,29] and during simulated cross-country skiing on roller skis [24,26–28]. But none compare the V1 and V2 freestyle skate techniques with the classic cross-country technique using roller skis during a graded exercise test. The results of this study showed that the energy costs of cross-country freestyle skating techniques are higher than those of the classic technique at comparable workloads. One vision of the 2020 XXIV Winter Olympics is to encourage at least 300 million Chinese youths and adults to engage in winter sports to attain healthy and active lifestyles and to promote nationwide physical fitness [41]. Achievement of this vision has the potential to make a substantial positive impact on the public's health. Knowing the energy cost of skiing and snowboarding provides information needed to promote these winter sports as health-enhancing, vigorous-intensity sporting activities and allows participants to realize gains in health and physical fitness.

Our study has some limitations. First, the Alpine skiing and snowboarding trials were performed in Northeast China in late March; the softer spring snow may have increased the friction between the base of the ski and the surface of the snow causing increased effort to complete the trials. Second, the majority of the skiers were male in the Alpine ski trial (11 males, 2 females) and no females were enrolled in the snowboarding trial, the limited female sample size limits the validity of the study's findings. Third, possible differences in equipment weights may cause deviation when measuring energy cost. Fourth, some participants fell during the Alpine skiing and snowboarding events, requiring them to start over. Further, the simulated cross-country skiing trials were performed on roller skis on a laboratory treadmill. The energy cost of cross-country skiing on snow may differ from the values obtained in this study, for which we set a maximum intensity to explain the difference. It is important to acquire data from actual cross-country skiing fields to facilitate more in-depth investigation and analysis. Last, the cross-country V1 and V2 freestyle skate techniques were used interchangeably during the treadmill trial. The ability to interchange V1 and V2 techniques may have affected the data by causing fluctuations in metabolic demand at different phases or by allowing participants to adopt individualized pacing strategies.

## 6. Conclusion

This study identified the energy cost of recreational Alpine skiing, snowboarding, and simulated cross-country skiing in young adult skiers residing in Northeast China. Performed on a downhill ski slope, Alpine skiing and snowboarding were classified as vigorous-intensity sport activities, with higher MET values for males in Alpine skiing than in snowboarding (7.90 ± 1.25 METs vs. 7.38 ± 2.55, respectively). The MET value for Alpine skiing in females was 7.10 ± 0.56. Simulated cross-country skiing was performed using roller skiing during a treadmill graded exercise test. RQ values indicated that the activities were moderate-to-vigorous intensity metabolic activities. This study not only quantifies the physiological demands of different skiing techniques but, more importantly, provides empirical, protocol-specific MET values. These findings address a data gap in the existing Adult Compendium of Physical Activities for skiing disciplines. By refining the metabolic costs associated with different techniques and intensities, this research offers a crucial empirical reference for exercise prescription, energy expenditure estimation, and the development of related equipment.
